# Characterizing the dispersion behavior of poly-atomic magnetic metamaterials

**DOI:** 10.1038/s41598-024-67248-7

**Published:** 2024-07-15

**Authors:** Connor Jenkins, Asimina Kiourti

**Affiliations:** ElectroScience Laboratory, Department of Electrical and Computer Engineering, The Ohio State University, Columbus, OH 43212 USA

**Keywords:** Electrical and electronic engineering, Electronic and spintronic devices

## Abstract

The propagation of magnetoinductive (MI) waves across magnetic metamaterials known as magnetoinductive waveguides (MIWs) has been an area of interest for many applications due to the flexible design and low-loss performance in challenging radio-frequency (RF) environments. Thus far, the dispersion behavior of MIWs has been limited to mono- and diatomic geometries. In this work, we present a recursive method to generate the dispersion equation for a general poly-atomic MIW. This recursive method greatly simplifies the ability to create closed-form dispersion equations for unique poly-atomic MIW geometries versus the previous method. To demonstrate, four MIW geometries that have been selected for their unique symmetries are analyzed using the recursive method. Using applicable simplifications brought on by the geometric symmetries, a closed-form dispersion equation is reported for each case. The equations are then validated numerically and show excellent agreement in all four cases. This work simultaneously aids in the further development of MIW theory and offers new avenues for MIW design in the presented dispersion equations.

## Introduction

The study of metamaterials, here defined as a composite material designed to achieve a desired permittivity and/or permeability, has a long history that was accelerated through observing negative permeability behavior in dielectric materials with periodically spaced metallic elements^1,2^. The study and use of metamaterials has since grown substantially due to the flexibility awarded in having tunable material electrical properties in applications such as absorber design, wireless power transfer, antenna design, and smart communication systems^3–6^.

While these applications take advantage of the interaction of electromagnetic waves with metamaterials, another field of interest are the waves these structures can generate and support themselves^7^. The focus of this work in particular is the magnetoinductive waveguide (MIW)—a magnetic metamaterial that is excited with a current that produces a traveling wave phenomenon^8,9^. While first formally described by Shamonina^8,9^, the idea of wave propagation supported by similar periodic structures extends back decades. For example, the band-pass behavior of one-dimensional lattices is discussed in great detail in^10^ and includes similar mathematical outcomes. Looking to common realized structures, resonant filters have likewise taken advantage of similar equivalent electric geometries to achieve the band-pass effect through inductive coupling for decades^11,12^. With this early history in mind, the last two decades have been an era of particular growth for the theory and applications of MIWs.

Thus far for MIWs, the dispersion equation relating the excitation frequency with a complex propagation constant has been defined in theory for mono-atomic (single resonant element per periodic unit) MIWs in 1-, 2-, and 3- dimensions and for di-atomic (two resonant elements per periodic unit) MIWs in 1-dimension^9,13^. In more unique geometric scenarios, the dispersion behavior of coalesced resonant elements has also been defined in detail along with di-atomic geometries that support both backward and forward waves^14,15^. Besides the wave guiding theory, the wave behavior has also been defined through lossless and lossy materials and in the presence of noise^16–19^. With this theory defined, several passive MI wave guiding devices have been created, such as phase shifters, resonators, power splitters, filters and transducers^20–23^. Despite the mature development of MIW technology, their use thus far has been limited. The four main challenges that MIWs face are (1) the complex and highly frequency-dependent matching impedance required to reduce reflections, (2) the related difficulty in designing well-matched transducers particularly for the di-atomic case, (3) relatively high path loss compared with traditional waveguides, and (4) their narrow band of operation. Despite these challenges, MIWs have been used as a valuable communication medium for a variety of applications such as in Magnetic Resonance Imaging systems^24–26^ or in traditionally challenging RF environments such as on the body^27–30^, underground^31–33^, or underwater^34,35^ where the magnetic-field-based nature of MIWs can be successfully leveraged. Outside of communications, the MIW operating principle has allowed for interesting applications in fields such as sub-wavelength imaging^36–38^, wireless power transfer^39–42^, and in a variety of sensing applications such as the localization of conductive materials^43–45^, defect estimation^46^, and localization in high frequency near-field communication systems^47,48^.

As previously discussed, the theoretical analysis of one-dimensional MIWs has thus far been limited to mono- and di-atomic MIWs^9,13^. The expansion from the one- to two-element case allowed for expanded bandwidths, two separable bands of operation, reduced path loss, and the supporting of both forward and backward waves. These benefits came at the cost of increased difficulty in impedance matching due to the number of elements that must be accounted for when synthesizing the matching impedance^13^ and an increased analysis complexity. In particular, the difficulty in transducer design for the di-atomic case has been the primary reason that they remain mostly unused in MIW applications. Despite the even further increased challenges in impedance matching, expanding to a general poly-atomic case (*K* resonant elements per periodic unit) is still of interest. The expansion would allow for up to *K* separable bands of operation and potential for even further reduced path loss and expanded bandwidths which would benefit many of the applications of MIWs described previously. One reason this expansion has yet to be realized in theory, besides the foreseen challenges in implementation and impedance matching, are the computational challenges facing the generation of the dispersion equations. The traditional analysis method relies on the determinant of the impedance matrix which grows in size as the square of the number of elements in the periodic unit (e.g., $K=3$ has 9 entries, $K=4$ has 16 entries, etc.) which becomes computationally intensive for large values of *K*. An alternative approach to this method for specific geometries is to examine the geometry as an infinite two-dimensional array as described by Shamonina^9^ and assume a standing wave solution in one direction. This method requires a strong working knowledge of 2D MIW arrays and also breaks down in the general case. So, while there exists interest in expanding the theory beyond the di-atomic case due to the potential for improved performance, it has not yet been explored due to the cumbersome nature of the traditional analytical method and the inability to generalize the truncated array method.

In this work, we create a novel recursive method to generate a dispersion equation for a general poly-atomic (*K* resonant elements per periodic unit) MIW through the use of the nearest neighbor approximation and coupled chain analysis. This method greatly simplifies the generation of new closed-form dispersion equations compared to traditional methods. Using this new recursive method, we generate closed-form dispersion equations for four geometries with unique symmetries and applicable simplifications, and then validate these equations numerically as both a proof-of-concept for the utility of the new method and an expansion of the current MIW design space.

## Results

### Derivation of recursive method

**Figure 1:**
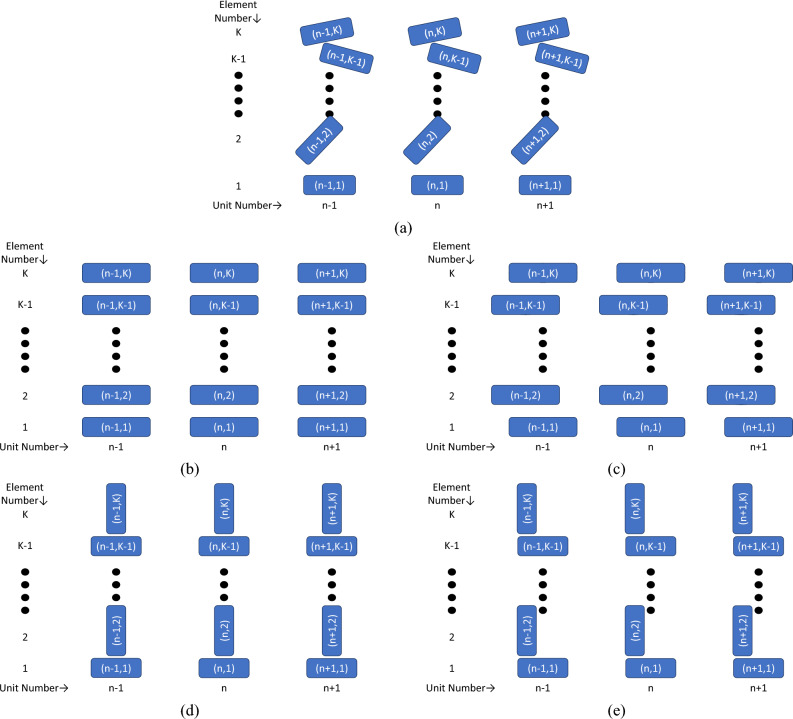
Diagram of poly-atomic MIWs with K elements per unit cell. Each rectangle represents a resonant element in a specific orientation. The elements are labeled by their element number in the periodic unit (*k*) and their unit number (*n*). Each element is identical and the direction of propagation is horizontal. (**a**) General case with all elements that share an element number having the same orientation as one another. (**b**) Case (1) with each element in the periodic unit aligned in orientation and position and with uniform spacing between elements within the periodic unit. (**c**) Case 2) where each element in the periodic unit is aligned in orientation and the spacing between elements within the periodic unit is uniform. An alternating shift between elements in the periodic unit is introduced. (**d**) Case (3) has each element in the periodic unit aligned in position and has the uniform spacing between elements in the periodic unit, but an alternating 90 degree rotation of elements is introduced. (**e**) Case (4) has uniform spacing within the periodic unit but an alternating rotation and shift of elements.

Figure 1a shows a truncated diagram of a general poly-atomic MIW. In this general scenario, we assume that each resonant element is identical (as is often the case), the MIW is periodic in the direction of propagation (i.e., the $n-1$th unit cell is identical to the *n*th unit cell and so forth), and that the MIW is infinite in length in the direction of propagation such that terminal reflections can be ignored. Additionally, we assume the coupling between non-nearest neighbor periodic units is significantly smaller than nearest neighbor coupling and can, thus, be ignored. Note that this is approximation is used only to simplify the presentation of the analytical work. We will show later on that expanding the method to include non-nearest neighbor interactions between periodic units is simple and follows from previous theory. As seen, the elements that make up each unit can be rotated at any angle (in 3D space) and can be shifted by up to $+/-$ half of the geometric period of the MIW relative to the first element which we select as the 0 reference point. Table 1 contains the dictionary for the various terms used throughout the derivation.

**Table 1:** Dictionary of mathematical symbols and their corresponding meaning for the derivation and application of the recursive method to generate closed-form dispersion equations.

Symbol	Parameter
$\omega$	Radial frequency
*R*	Resistance of element
*L*	Self-inductance of element
*C*	Capacitance of element
*Z*	Characteristic impedance of element
$M_{(n,k),(u,q)}$	Mutual inductance between elements (n,k) and (u,q)
$Z_{(n,k),(u,q)}$	Mutual impedance between elements (n,k) and (u,q)
$I_{n,k}$	Current on loop (n,k)
$I_k$	Current constant on *k*th elements
$\gamma$	Complex propagation constant
$\alpha$	Attenuation constant
$\beta$	Phase constant
*p*	Geometric period of the MIW
*K*	Number of elements per periodic unit
$\varvec{Z}_K$	Impedance matrix of *K*-element per period MIW
$\varvec{V}_K$	Voltage vector of *K*-element per period MIW
$\varvec{I}_K$	Current vector of *K*-element per period MIW

The proposed derivation of the relationship between the complex propagation constant, $\gamma$, and the radial frequency, $\omega$, for a poly-atomic MIW follows a generalized form of the coupled chain analysis conducted for the two-element case^13^. We begin by invoking Kirchoff’s Voltage Law on each element in the *n*th unit, assuming that the non-nearest neighbor coupling in the direction of propagation is negligible. This generates three types of expressions due to the finite number of elements: (1) an expression for the 1st element, (2) an expression for any *k*th element, and (3) an expression for the *K*th element. For illustrative purposes, we will focus just on the 1st element, with all others following a similar procedure and form. Assuming no external excitation on the *n*th loop:

1 $$\begin{aligned} 0 =I_{n,1}Z + I_{n-1,1}Z_{(n-1,1),(n,1)} + I_{n+1,1}Z_{(n+1,1),(n,1)} + \sum _{i=2}^{K}I_{n,i}Z_{(n,i),(n,1)} + I_{n-1,i}Z_{(n-1,i),(n,1)} + I_{n+1,i}Z_{(n+1,i),(n,1)} \end{aligned}$$

We then look for wave solutions of the form $I_{n,k}=I_ke^{-nj\gamma p}$ which corresponds to a traveling wave in the positive *n* direction with $\gamma = \beta - j\alpha$. After simplification, we reach the following form for the first element:

2 $$\begin{aligned} 0 =I_1(Z+2Z_1cos(\gamma p)) + \sum _{i=2}^{K}I_i(Z_{(n,i),(n,1)} + e^{j\gamma p}Z_{(n-1,i),(n,1)} + e^{-j\gamma p}Z_{(n+1,i),(n,1)} \end{aligned}$$

With $Z_{(n+1,k),(n,k)}=Z_{(n-1,k),(n,k)}=Z_k$. Similar expressions are created for the *k*th and *K*th elements as well.

From these expressions, we convert the system of equations to matrix form such that $\varvec{V}_K = \varvec{Z}_K\varvec{I}_K$. Here, $\varvec{V}_K$ is a $K\times 1$ 0 vector and $\varvec{I}_K$ contains the *K* current constants noted previously. To simplify the notation of the impedance matrix, we set $a_i=Z+2Z_icos(\gamma p)$ and $b_{ik}=Z_{(n,k),(n,i)}+ e^{j\gamma p}Z_{(n-1,k),(n,i)} + e^{-j\gamma p}Z_{(n+1,k),(n,i)}$. Thus the impedance matrix for a general poly-atomic MIW is:

3 $$\begin{aligned} \varvec{Z}_K= \begin{bmatrix} a_1 &{}\quad b_{12} &{}\quad \dots &{}\quad b_{1K}\\ b_{21} &{}\quad a_2 &{}\quad \ddots &{}\quad \vdots \\ \vdots &{}\quad \ddots &{}\quad \ddots &{}\quad b_{K-1,K}\\ b_{K1} &{}\quad \dots &{}\quad b_{K,K-1} &{}\quad a_K\\ \end{bmatrix} \end{aligned}$$

To generate non-trivial solutions to the equation $\varvec{V}_K=\varvec{Z}_K\varvec{I}_K$, we require that $|\varvec{Z}_K|=0$. This condition relates the propagation constant ($\gamma$) and the radial frequency ($\omega$), thus creating a dispersion equation for any MIW. The problem with generalizing this condition is that each element in $\varvec{Z}_K$ is unique, i.e., generalizing the condition means generalizing the determinant. This is a problem that is already well studied and has several solutions. For example, we can use the Laplace expansion to generate the determinants through the cofactor matrix. As previously discussed, this is the typical approach used to generate closed-form dispersion equations for any MIW^9,13^. Our goal is to create a simplified method of generating these dispersion equations and to do so, we must enforce further simplifications.

To simplify the expression, we further apply the nearest neighbor approximation such that we only account for nearest neighbor coupling in the direction of propagation and within each periodic unit. Physically, this means that the elements within our periodic unit are weakly coupled to one another such that the coupling between elements (*n*, *k*) and $(n,k\pm 1)$ is much larger than the coupling between elements (*n*, *k*) and $(n,k\pm 2)$. Instead of working through the derivation process again, we can simply see that this approximation leads to all $b_{ik} = 0$ for $|i-k|>1$. This leads to a new impedance matrix:

4 $$\begin{aligned} \varvec{Z}_{K}= \begin{bmatrix} a_1 &{}\quad b_{12} &{}\quad 0 &{}\quad \dots &{}\quad 0\\ b_{21} &{}\quad a_2 &{}\quad b_{23}&{}\quad \ddots &{}\quad \vdots \\ 0 &{}\quad b_{32} &{}\quad a_3 &{}\quad \ddots &{}\quad 0\\ \vdots &{}\quad \ddots &{}\quad \ddots &{}\quad \ddots &{}\quad b_{K-1,K}\\ 0 &{} \quad \dots &{}\quad 0 &{}\quad b_{K,K-1} &{}\quad a_K\\ \end{bmatrix} \end{aligned}$$

With many 0 terms, there is now potential to further generalize the determinant condition beyond the typical approaches. Through the use of the Laplace expansion, the first few dispersion equations are:

5 $$\begin{aligned} K=1\rightarrow 0&= a_1 \end{aligned}$$

6 $$\begin{aligned} K=2\rightarrow 0&= a_2(a_1) - b_{21}b_{12} \end{aligned}$$

7 $$\begin{aligned} K=3\rightarrow 0&= a_3(a_1a_2 -b_{21}b_{12})-b_{32}b_{23}(a_1) \end{aligned}$$

From Eqs. (5)–(7), a clear pattern emerges that does continue to higher values of *K*. For a general poly-atomic MIW, we can now determine the dispersion equation recursively as:

8 $$\begin{aligned} |\varvec{Z}_{K}|=a_K|\varvec{Z}_{K-1}| - b_{K,K-1}b_{K-1,K}|\varvec{Z}_{K-2}|=0 \end{aligned}$$

Where $|\varvec{Z}_K|$ is the determinant of matrix $\varvec{Z}_K$. Although this recursive method will generate a general dispersion equation for any value of *K*, we can also use this method to examine simplified geometries to generate a closed-form expression for their dispersion equations. We will examine four (4) distinct cases: Case (1) Fully Uniform Array, Case (2) Uniform Array with Alternating Shift, Case (3) Uniform Array with Alternating Rotation, Case (4) Uniform Array with Alternating Rotation and Shift. While we will maintain the nearest neighbor approximation between periodic units for each Case to simplify the analysis, this recursive method can still be used in the case of significant non-nearest neighbor coupling between periodic units with an extension of the $a_i$ and $b_{ik}$ definitions.

### Application

For Case (1), we take the restriction to the extreme and enforce rotation and position alignment and uniform spacing of all elements as shown in Fig. 1b. Mathematically, this leads to $Z_k=Z_1$, $Z_{(n,k+1)(n,k)}=Z_{(n,2)(n,1)}$ and $Z_{(n+1,k+1)(n,k)}=Z_{(n-1,k+1)(n,k)}=Z_{(n+1,2)(n,1)}=Z_{(n-1,2)(n,1)}=Z_{(n\pm 1,2)(n,1)}$. In terms of the matrix terms, we now have $a_1=a_2=\dots =a_K=a=Z+2Z_1cos(\gamma p)$ and $b_{ik}=b_{ki}=b=Z_{(n,2),(n,1)}+2Z_{(n\pm 1,2),(n,1)}cos(\gamma p)$. With these new expressions, we again look at the first few dispersion equations:

9 $$\begin{aligned} K=1\rightarrow 0&= a \end{aligned}$$

10 $$\begin{aligned} K=2\rightarrow 0&= a^2 - b^2 \end{aligned}$$

11 $$\begin{aligned} K=3\rightarrow 0&= a^3 - 2ab^2 \end{aligned}$$

12 $$\begin{aligned} K=4\rightarrow 0&= a^4 - 3a^2b^2 + b^4 \end{aligned}$$

13 $$\begin{aligned} K=5\rightarrow 0&= a^5 - 4a^3b^2 + 3ab^4 \end{aligned}$$

14 $$\begin{aligned} K=6\rightarrow 0&= a^6 - 5a^4b^2 + 6a^2b^4-b^6 \end{aligned}$$

With Eqs. (9)–(14) completed, a pattern emerges as a result of the symmetry. Based on this, the closed-form dispersion equation for a poly-atomic MIW with identical, aligned elements and uniform spacing within the periodic unit is:

15 $$\begin{aligned} 0 = a^K + \sum _{s=1}^{\left\lfloor {K/2}\right\rfloor }(-1)^s \left( \begin{array}{c} K-s\\ s \end{array}\right) \ a^{K-2s}b^{2s} \end{aligned}$$

From this expression, the polynomial nature of the dispersion equations for MIWs becomes very clear. The introduction of additional elements in each periodic unit leads to additional solutions to the polynomial expression. In turn, this leads to additional propagation constants such that a poly-atomic MIW may have up to *K* separable frequency bands of operation.

For Case (2), we generalize the expression further by relaxing some of the uniformity requirements. First, we allow for a periodic shift of every other element in the unit as shown in Fig. 1c. We still have $Z_k=Z_1$ and $Z_{(n,k+1)(n,k)}=Z_{(n,2)(n,1)}$, such that $a_1=a_2=\dots =a_K=a$, but we have different symmetries for the diagonal terms depending on whether *k* is even or odd. For even *k*, we have:

16 $$\begin{aligned} Z_{(n+1,k+1),(n,k)}&=Z_{(n+1,k-1),(n,k)}=Z_{(n-1,2),(n,1)} \end{aligned}$$

17 $$\begin{aligned} Z_{(n-1,k+1),(n,k)}&=Z_{(n-1,k-1),(n,k)}=Z_{(n+1,2),(n,1)} \end{aligned}$$

For odd *k*, the symmetry dictates:

18 $$\begin{aligned} Z_{(n+1,k+1),(n,k)}&=Z_{(n+1,k-1),(n,k)}=Z_{(n+1,2),(n,1)}, \end{aligned}$$

19 $$\begin{aligned} Z_{(n-1,k+1),(n,k)}&=Z_{(n-1,k-1),(n,k)}=Z_{(n-1,2),(n,1)} \end{aligned}$$

This gives us two off-diagonal terms and one on-diagonal term: $a=Z+Z_1cos(\gamma p)$, $b_1=Z_{(n,2),(n,1)} + e^{j\gamma p}Z_{(n-1,2),(n,1)} +e^{-j\gamma p}Z_{(n+1,2),(n,1)}$, and $b_2=Z_{(n,2),(n,1)} + e^{j\gamma p}Z_{(n+1,2),(n,1)} +e^{-j\gamma p}Z_{(n-1,2),(n,1)}$. Following the same process with the dispersion equations, a similar pattern emerges which gives the following general dispersion equation:

20 $$\begin{aligned} 0 = a^K + \sum _{s=1}^{\left\lfloor {K/2}\right\rfloor }(-1)^s \left( \begin{array}{c} K-s\\ s \end{array}\right) \ a^{K-2s}(b_1b_2)^{s} \end{aligned}$$

The equation is very similar to Eq. (15) as anticipated and also simplifies to Eq. (15) when the geometric shift is 0 or $\pm p/2$, such that $b_1=b_2$.

Case (3) is the dual of Case (2) in that we maintain the off-diagonal symmetry while introducing periodicity in the on-diagonal entries from the initial case. This can be easily accomplished through an alternating 90-degree rotation between aligned elements in each unit, as shown in Fig. 1d. By rotating the elements, we now have an additional $Z_1=Z_3=Z_5\dots$ and $Z_2=Z_4=Z_6=\dots$. This implies the two on-diagonal terms $a_1=Z + 2Z_1cos(\gamma p)$ and $a_2=Z+2Z_2cos(\gamma p)$. Examining Eq. (8), we see that we now have separate expressions when *K* is even or odd. Because of the center alignment of the elements, we still maintain $b_{ik}=b_{ki}=b=Z_{(n,2),(n,1)}+2Z_{(n\pm 1,2),(n,1)}cos(\gamma p)$. The new recursive relation is:

21 $$\begin{aligned} |\varvec{Z}_{K;Odd}|&= a_1|\varvec{Z}_{K-1}| - b^2|\varvec{Z}_{K-2}|=0 \end{aligned}$$

22 $$\begin{aligned} |\varvec{Z}_{K;Even}|&= a_2|\varvec{Z}_{K-1}| - b^2|\varvec{Z}_{K-2}|=0 \end{aligned}$$

While the even/odd nature of the relationship complicates the analysis, the same procedure can be followed to generate a closed-form solution that is similar to both Eqs. (15) and (20):

23 $$\begin{aligned} 0 = a_1^{\big \lceil \frac{K}{2}\big \rceil }a_2^{\big \lfloor \frac{K}{2}\big \rfloor } + \sum _{s=1}^{\big \lfloor {K/2}\big \rfloor }(-1)^s \left( \begin{array}{c} K-s\\ s \end{array}\right) \ a_1^{\big \lceil \frac{K-2s}{2}\big \rceil }a_2^{\big \lfloor \frac{K-2s}{2}\big \rfloor }(b)^{2s} \end{aligned}$$

It is important to note that by introducing a rotated element into the unit cell, there is now the possibility of both forward and backward waves propagating on the structure. This phenomenon has been explored in depth in previous literature^13^.

Finally, in Case (4), we combine both Case (2) and (3) by introducing a periodic shift and 90-degree rotation within the unit cell, as shown in Fig. 1e. Without further analysis, it is clear that the symmetries will include a combination of the previous two cases such that

24 $$\begin{aligned} a_1&=Z + 2Z_1cos(\gamma p) \end{aligned}$$

25 $$\begin{aligned} a_2&=Z + 2Z_2cos(\gamma p) \end{aligned}$$

26 $$\begin{aligned} b_1&=Z_{(n,2),(n,1)} +e^{j\gamma p}Z_{(n-1,2),(n,1)} +e^{-j\gamma p}Z_{(n+1,2),(n,1)} \end{aligned}$$

27 $$\begin{aligned} b_2&=Z_{(n,2),(n,1)} +e^{j\gamma p}Z_{(n+1,2),(n,1)} +e^{-j\gamma p}Z_{(n-1,2),(n,1)} \end{aligned}$$

are the only terms in the impedance matrix. Following the derivations of Eqs. (20) and (23), this leads to a dispersion equation of the form:

28 $$\begin{aligned} 0 = a_1^{\big \lceil \frac{K}{2}\big \rceil }a_2^{\big \lfloor \frac{K}{2}\big \rfloor } + \sum _{s=1}^{\big \lfloor {K/2}\big \rfloor }(-1)^s \left( \begin{array}{c} K-s\\ s \end{array}\right) \ a_1^{\big \lceil \frac{K-2s}{2}\big \rceil }a_2^{\big \lfloor \frac{K-2s}{2}\big \rfloor }(b_1b_2)^{s} \end{aligned}$$

As previously mentioned, the presented dispersion equations in Eqs. (15), (20), (23), and (28) rely on the nearest neighbor approximation in all directions for each resonant element. While we cannot relax the approximation within the unit cell while maintaining the form of the expressions, we can relax the approximation between unit cells with relative ease. As an example, when considering *U* nearest neighbors between unit cells of the uniform and aligned MIW shown in Fig. 1b, the dispersion equation remains the same, just with new definitions of *a* and *b*.

29 $$\begin{aligned} a&=Z + \sum _{u=1}^U2Z_{(n\pm u,1)}cos(\gamma p) \end{aligned}$$

30 $$\begin{aligned} b&=Z_{(n,2),(n,1)}+\sum _{u=1}^U2Z_{(n\pm u,2),(n,1)}cos(\gamma p) \end{aligned}$$

This foundation can be easily applied for each of the other cases explored throughout this work.

### Validation

**Table 2:** All mutual inductance values used in the validation of Case (1)–Case (4).

	Case (1) (nH)	Case (2) (nH)	Case (3) (nH)	Case (4) (nH)
$M_{(n,2),(n,1)}$	62.8	45.3	0.0007	− 12.0
$M_{(n\pm 1,1),(n,1)}$	− 30.3	− 30.3	− 30.3	− 30.3
$M_{(n\pm 1,2),(n,2)}$	− 30.3	− 30.3	10.8	10.8
$M_{(n+1,2),(n,1)}$	− 11.3	1.1	− 7.7	− 13.4
$M_{(n-1,2),(n,1)}$	− 11.3	− 7.7	− 7.7	− 4.7

To validate each analytical equation, we use the CST Studio finite-element method, frequency domain, 3D full-wave electromagnetic solver^49^. This method was selected to demonstrate that the relatively simple dispersion equations can predict MIW performance with a high-level of accuracy despite not accounting for effects such as non-nearest neighbor coupling, electric coupling, and radiation that are present in both the full-wave simulation and in reality. While experimental validation is outside the scope of this work, we note strong agreement between similar simulations and experiments have been previously demonstrated^27,28,30^. As such, the presented simulation results are expected to be a good approximation of experimental results.

For each MIW in each Case, the resonant elements are 9.1 $\times$ 3.5 cm copper rectangular loops made of 30 American Wire Gauge wire and loaded with 56 pF capacitors to achieve resonance at 41.3 MHz. This design corresponds to equivalent circuit parameters of $R=0.92$ $\Omega$, $L=260.3$ *nH*, and $C=56$ *pF*. The physical gap between units in all scenarios is set at 0.25 cm and the loops are oriented in such a way that the overall geometric period is 3.75 cm. The distance between elements is set to 0.75 cm. Both of these distances were selected to reduce the effect of non-nearest neighbor coupling to meet our base assumptions for each equation. Table 2 contains the appropriate mutual inductance values for each Case.

To simulate each Case in CST Studio, we truncate the MIWs to 11 units, place the structure in free space, and excite the first element in the first unit with a 0.5 W sinusoidal signal. S-Parameters are then generated using a reference impedance of 50 $\Omega$. Because of the selected simulation method and lack of a mode-selective and well-matched transducer for poly-atomic MIWs, the wave modes cannot be easily separated. As such, the S-Parameters are presented separately from the calculated attenuation and phase constants. Instead of directly comparing the analytical model results to equivalent propagation constants calculated from the S-Parameters, the bandwidth and loss predicted by the analytical model will be compared to the performance shown in the corresponding S-Parameters. Because the analytical model does not account for reflections, the $|S_{21}|$ results will be the primary tool for comparison. Despite the focus of this work on the propagation behavior, $|S_{11}|$ results are also included to highlight the future need of effective transducer design for the real-world application of poly-atomic MIWs.

**Figure 2:**
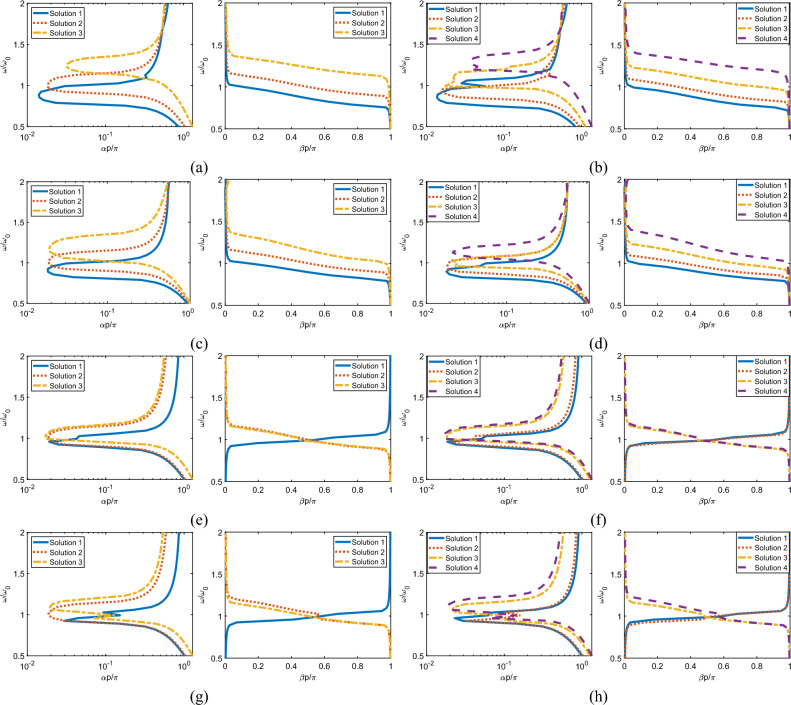
Frequency vs complex propagation constant results. Left image is imaginary attenuation constant ($\alpha$) and the right is the real phase constant ($\beta$). (**a, b**) Case (1) with uniform spacing, orientation and position in the periodic unit with $K=3$ and $K=4$ respectively. (**c, d**) Case (2) $K=3$ and $K=4$ with uniform orientation and spacing but alternating shift in the periodic unit. (**e, f**) Case (3) with alternating 90 degree rotations between aligned elements in the periodic unit with uniform spacing. $K=3$ and $K=4$ for (**e**) and (**f**). (**g, h**) Case (4) with alternating shifts and 90 degree rotations between elements in the periodic unit with uniform spacing. $K=3$ for (**g**) and $K=4$ for (**h**).

**Figure 3:**
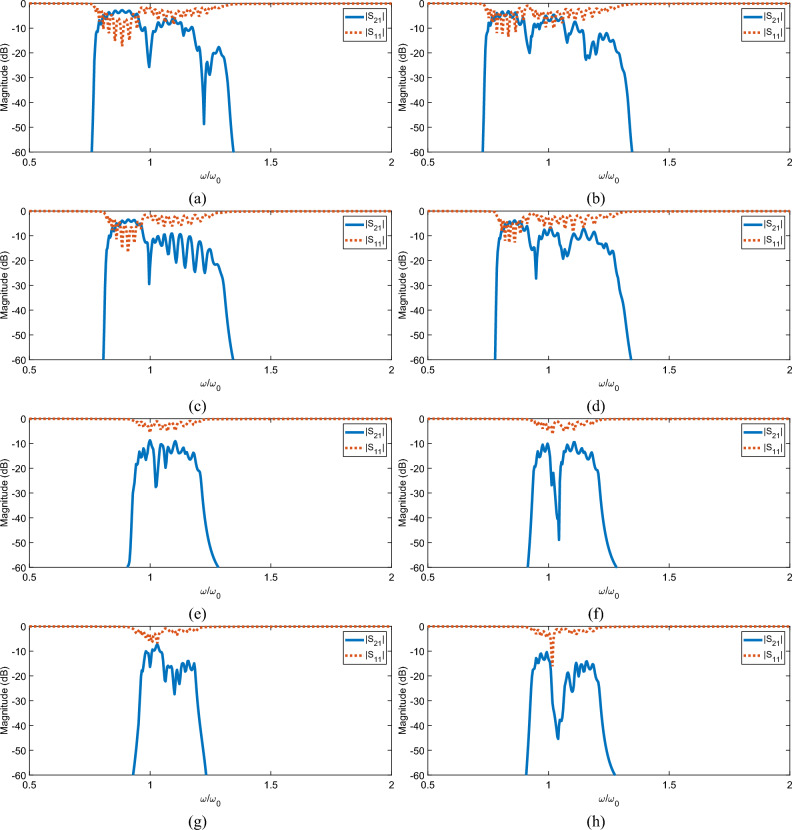
S-Parameter versus normalized frequency results. $|S_{11}|$ results are shown with dashed lines and $|S_{21}|$ results are shown with solid lines. (**a, b**) Case (1) with uniform spacing, orientation and position in the periodic unit with $K=3$ for for **(a)** and $K=4$ for (**b**) respectively. (**c, d**) Case (2) $K=3$ and $K=4$ with uniform orientation and spacing but alternating shift in the periodic unit. (**e, f**) Case (3) with alternating 90 degree rotations between aligned elements in the periodic unit with uniform spacing. $K=3$ and $K=4$ for **(e)** and (**f**). (**g, h**) Case (4) with alternating shifts and 90 degree rotations between elements in the periodic unit with uniform spacing. $K=3$ for (**g**) and $K=4$ for (**h**).

Case (1), as shown in Fig. 1b with the dispersion equation defined in Eq. (15), is analyzed for the $K=3$ and $K=4$ cases in Fig. 2a,b. Looking at the attenuation constants ($\alpha$), we first see the expected *K* separate solutions from the analytical model. For both $K=3$ and $K=4$, the first solution corresponds to the lowest attenuation across the examined frequency range. Figure 3a,b show the corresponding S-Parameter results. The $|S_{21}|$ results clearly show the same *K* separated passbands with relatively large nulls separating the bands. Additionally, the loss increases per band as the frequency increases for each case in both the S-Parameter and attenuation constant results.

For the phase constant ($\beta$), the bandwidth of each solution is defined by the frequencies where the derivative of the phase constant with respect to frequency is nonzero (i.e., exhibits a non-zero group velocity). For the S-Parameter results, the bandwidth definition is not as clear. We will define the number of bands and their corresponding bandwidths with the following procedure: (1) determine the value and frequency of the minimum attenuation, (2) from this point, increase and decrease the frequency until the attenuation is 20 dB less than the minimum attenuation found in (1) and set these two frequency points as the high and low-frequency cutoffs for the current band. (3) repeat steps (1) and (2), removing the previous band from the analysis. This process repeats a maximum of *K* times, corresponding to the *K* possible solutions. In the $K=3$ scenario for Case (1), the analytical results predict three passbands with the corresponding fractional bandwidths (with respect to the resonant frequency): 0.27, 0.30, and 0.30. Following our procedure, the S-Parameter results indicate fractional bandwidths of 0.22, 0.22, and 0.10 for each respective passband. While these bandwidths are significantly smaller than the analytical results, the analytical values do not account for the significant overlap in frequency between each solution. Instead we determine the overall bandwidth of the structure regardless of mode for the analytical results and compare this to the overall bandwidth of the simulated results. The analytical model predicts an overall bandwidth of 0.64 while the simulated results have an overall bandwidth of 0.56. This slight decrease in bandwidth is expected as the -20 dB definition of bandwidth used for the simulated results is stricter than the definition used in the analytical results which allows for extremely high levels of loss at the edges of the bands. Increasing *K* to 4 leads to similar results with the analytical model predicting bandwidths of 0.30, 0.30, 0.30 and 0.27 for each band and an overall bandwidth of 0.71 while the simulated results have bandwidths of 0.18, 0.16, 0.08, and 0.17 with an overall bandwidth of 0.58.

For Case (2), we introduce a quarter period (0.9325 cm) shift in every other element in the periodic unit. The analytical results of this second case, as shown in Fig. 1c that has a dispersion equation defined by Eq. (20), are shown in Fig. 2c,d and the simulated results are in Fig. 3c,d. In the $K=3$ scenario, only the first analytical passband is well represented by the simulated results while the upper bands are assimilated into a single passband due to large fluctuations in loss after the resonant frequency. This problem does not persist when *K* is increased to 4. Further examining the attenuation results, we see that the simulation upper frequency cutoff is decreased relative to the analytical model for both $K=3$ and $K=4$. This difference is likely caused by stronger relative coupling between non-nearest neighbors within the periodic unit when compared to the first case. By shifting every other element, the coupling between the *k*th and $k+1$th elements is decreased while the coupling between the *k*th and $k+2$th elements is unchanged. This decreases the validity of our base assumption that non-nearest neighbor interactions are negligible.

For the phase constants, the $K=3$ analytical model predicts passband with bandwidths 0.24, 0.30, 0.37 and an overall bandwidth of 0.58. Similarly, the $K=4$ model predicts bandwidths of 0.27, 0.27, 0.34, 0.37 and an overall bandwidth of 0.64. The separation between bands in the simulated results is poor as anticipated by the significant overlap between solutions in the analytical model as such the individual passband bandwidths are not meaningful. The overall bandwidth for both $K=3$ and $K=4$ align well with the analytical results, at 0.49 and 0.51 respectively with the primary decrease in bandwidth relative to the model coming from the decrease in upper frequency cutoff as previously described.

For Case (3), we rotate every other element in the periodic unit by 90 degrees while maintaining uniformity in center alignment. This implies both positive and negative mutual coupling between the periodic units, which introduces both forward and backward waves as previously discussed. Figure 2e,f shows the results for this case based on the dispersion equation presented in Eq. (23) and Fig. 3e,f contain the simulated results.

We see strong agreement for the $K=3$ and $K=4$ attenuation constants, with a slight discrepancy in the center of the passband where both simulation results present a slight increase in attenuation rather than the expected smooth behavior shown in the analytical model results. This is likely caused by non-nearest neighbor coupling within the periodic unit being significantly stronger than the nearest neighbor coupling within the periodic unit due to the 90 degree rotation. By placing the elements perpendicular to one another while maintaining their center alignment, we theoretically set the mutual coupling value between them to 0. As such, any non-zero coupling between non-nearest neighbor will have more impact on the results than other scenarios.

For the phase constant, we see the expected forward and backward waves in the analytical solutions. This is demonstrated by both negative and positive sloped curves across the phase constant. Regarding each passband, we have bandwidths of 0.24, 0.34, and 0.24 for the $K=3$ case and bandwidths of 0.20, 0.27, 0.24, and 0.24 for the $K=4$ case. As the phase constants lie essentially across identical frequencies, the overall predicted bandwidths of the structures are very close to the bandwidths of the individual solutions, at 0.34 for both cases. Likewise, the overall bandwidths in the simulated results are the same for both cases, at 0.28 which shows overall strong agreement in bandwidth between simulated and analytical results.

To model Case (4), we start at the geometry of Case (3) and shift the alternating rotated elements by a quarter period relative to the first element in the direction of propagation. This scenario is shown in Fig. 1e and modeled by Eq. (28). The analytical results are presented in Fig. 2g,h and simulated results in Fig. 3g,h.

As seen, the dispersive behavior is similar to Case (3) for both $K=3$ and $K=4$. This is because of the relatively weak coupling between adjacent elements in the periodic unit that is maintained by only shifting the elements by a small distance. Comparing to the simulation, we also see a very similar level of agreement for both $K=3$ and $K=4$. Again, the simulated attenuation constant is nearly identical to the analytical solutions, besides an increase in attenuation in the center of the passband. We can again explain this by the effects of non-nearest neighbor coupling.

For the phase constants, we again see strong agreement in the overall bandwidth between the simulated and analytical results for both $K=3$ and $K=4$ The model predicts an overall bandwidth of 0.37 for both $K=3$ and $K=4$ while the simulated results show overall bandwidths of 0.24 and 0.29 respectively. The larger difference in bandwidth for the $K=3$ case is primarily caused by a significant decrease in upper frequency cutoff compared with the $K=4$ case.

## Conclusion

In this work, we have significantly expanded the MI wave guiding theory by defining a new recursive method of determining the dispersion equation for a general poly-atomic (*K* resonant elements per periodic unit) MIW. This recursive method relies on the validity of the nearest neighbor approximation within the periodic unit but can easily be expanded to include higher order coupling effects in the direction of propagation. Using this method, four cases were examined, each with unique symmetries and applicable simplifications. For each case, a closed-form dispersion equation was generated using the recursive method to greatly reduce the complexity of analysis. Each case was then validated through the use of simulation results for $K=3$ and $K=4$. Results showed strong agreement between the analytical and numerical attenuation and phase behaviors over frequency. Discrepancies in results are likely caused by non-nearest neighbor coupling effects within the periodic unit that are not accounted for in the new recursive method and subsequently generated closed-form equations.

Through the new dispersion equations and recursive dispersion equation generation method, the MIW design space has been expanded significantly. In particular, the theory was previously limited to mono- or di-atomic MIWs but has now been expanded to any number of elements per period. The new closed-form equations expand the specific theory of MI wave propagation while the new method expands the tools available to those interested in specific and unique geometries for their applications such as imaging, communications, or sensing. While these new ideas rely on the validity of the nearest neighbor approximation within the periodic unit, they still serve as a useful approximation for cases where non-nearest neighbor coupling is significant, particularly for large value of *K* where the typical analysis becomes cumbersome. Future work will include validating the results presented here experimentally, expanding the analysis to include the synthesis of the required terminal impedance to reduce reflections in poly-atomic MIWs, and the design of mode-selective, well-matched transducers for di-atomic and poly-atomic MIWs.

## Data Availability

The data supporting the findings of this paper are available upon reasonable request to the corresponding author, C.J..
